# FBXW7 deletion contributes to lung tumor development and confers resistance to gefitinib therapy

**DOI:** 10.1002/1878-0261.12200

**Published:** 2018-05-09

**Authors:** Yi Xiao, Chunli Yin, Yuli Wang, Hanlin Lv, Wenqing Wang, Yurong Huang, Jesus Perez‐Losada, Antoine M. Snijders, Jian‐Hua Mao, Pengju Zhang

**Affiliations:** ^1^ Department of Biochemistry and Molecular Biology Shandong University School of Basic Medical Sciences Jinan China; ^2^ Department of Clinical Laboratory The Second Hospital of Shandong University Jinan China; ^3^ Biological Systems and Engineering Division Lawrence Berkeley National Laboratory CA USA; ^4^ Instituto de Biología Molecular y Celular del Cáncer (IBMCC) Instituto Mixto Universidad de Salamanca/CSIC IBSAL Salamanca Spain

**Keywords:** EGFR‐TKI, FBXW7, gefitinib, NSCLC

## Abstract

Gefitinib, an epidermal growth factor receptor–tyrosine kinase inhibitor (EGFR‐TKI), is an effective treatment for non‐small‐cell lung cancer (NSCLC) with EGFR activating mutations, but inevitably, the clinical efficacy is impeded by the emergence of acquired resistance. The tumor suppressor gene FBXW7 modulates chemosensitivity in various human cancers. However, its role in EGFR‐TKI therapy in NSCLC has not been well studied. Here, we demonstrate that the mice with deficient *Fbxw7* have greater susceptibility to urethane‐induced lung tumor development. Through analysis of The Cancer Genome Atlas data, we show that deletion of *FBXW7* occurs in 30.9% of lung adenocarcinomas and 63.5% of lung squamous cell carcinomas, which significantly leads to decrease in FBXW7 mRNA expression. The reduction in FBXW7 mRNA level is associated with poor overall survival in lung cancer patients. FBXW7 knockdown dramatically promotes epithelial–mesenchymal transition, migration, and invasion in NSCLC cells. Moreover, with silenced FBXW7, EGFR‐TKI‐sensitive cells become resistant to gefitinib, which is reversed by the mammalian target of rapamycin inhibitor, rapamycin. Furthermore, xenograft mouse model studies show that FBXW7 knockdown enhances tumorigenesis and resistance to gefitinib. Combination of gefitinib with rapamycin treatment suppresses tumor formation of gefitinib‐resistant (GR) FBXW7‐knockdown cells. In conclusion, our findings suggest that loss of FBXW7 promotes NSCLC progression as well as gefitinib resistance and combination of gefitinib and rapamycin may provide an effective therapy for GR NSCLC.

**Abbreviations**
ACs: adenocarcinomas
EGFR: epidermal growth factor receptor
EGFR‐TKI: epidermal growth factor receptor–tyrosine kinase inhibitor
EMT: epithelial–mesenchymal transition
FBXW7: F‐box and WD40 domain protein 7
GR: gefitinib resistant
mTOR: mammalian target of rapamycin
NSCLC: non‐small‐cell lung cancer
SCCs: squamous cell carcinomas

## 1 Introduction

Lung cancer accounts for the most common cause of cancer death and has the highest incidence rates among all malignant tumors worldwide. Non‐small‐cell lung cancer (NSCLC) accounts for approximately 85% of lung cancers and carries a 5‐year survival rate of only 15%, which is even lower when accompanied by metastasis. Although surgical resection remains the most consistent and successful option for patients diagnosed with lung cancer, the feasibility is invariably limited because patients’ surgical tolerance and their cancer stage might not be optimal (Molina *et al*., 2008; Siegel *et al*., 2017). Therefore, more effective treatments should be explored for NSCLC patients who are not suitable for surgery. Over the past decade, many novel therapeutic targets have been identified for lung cancer treatment. The detection of mutations in the epidermal growth factor receptor (EGFR) gene has brought encouraging improvements in treatment of advanced NSCLC due to the efficacy of EGFR–tyrosine kinase inhibitors (EGFR‐TKIs) (Fukuoka *et al*., 2011; Wood *et al*., 2015).

Epidermal growth factor receptor is highly expressed in a variety of solid tumors, including NSCLC, and its activation has been shown to promote processes involved in tumor cell proliferation, angiogenesis, invasion, metastasis, and inhibition of apoptosis (Vansteenkiste, 2004). One of the mechanisms of increased EGFR signaling is activating EGFR mutations. Overall, EGFR exon 19 deletion mutations and the point mutation of L858R constitute about 90% of all EGFR activating mutations and are termed ‘classical’ activating mutations. Mutant kinases demonstrate a reduced affinity for ATP, which provides a molecular explanation for the increased sensitivity to TKI. Gefitinib, as the first‐line EGFR‐TKI, provides significant clinical benefits in NSCLC patients, yet acquired resistance occurs in virtually all NSCLC tumors that initially respond to it (Gazdar, 2009). Hence, dissecting the molecular mechanisms of gefitinib resistance is of great necessity.

FBXW7 (F‐box and WD40 domain protein 7) functions as a substrate recognition subunit of the SKP1‐CUL1‐F‐box protein (SCF) E3 ubiquitin ligase complex, which plays a central role in cell division, growth, and differentiation through targeting well‐known oncoproteins, including mammalian target of rapamycin (mTOR), c‐Myc, c‐Jun, and Notch (Cheng and Li, 2012; Mao *et al*., 2008; Welcker and Clurman, 2008). Generally, FBXW7 is regarded as a tumor suppressor and its deletion or mutation has been reported in many different types of cancers (Akhoondi *et al*., 2007; Mao *et al*., 2004; Rajagopalan *et al*., 2004; Spruck *et al*., 2002). Even though many studies have implied that FBXW7 mediates chemotherapeutic sensitivity, relatively few studies focused on the associations between FBXW7 and drug resistance in NSCLC (Yokobori *et al*., 2014; Yu *et al*., 2013). Furthermore, the mechanisms relating to how FBXW7 executes its role as a tumor suppressor to enhance chemosensitivity in NSCLC are poorly elucidated.

In this study, we investigate whether FBXW7 plays an important role in lung tumor development and whether FBXW7 depletion affects outcome of gefitinib therapy.

## 2 Materials and methods

### 2.1 Mice and tumor induction

Wild‐type and *Fbxw7* heterozygous (*Fbxw7*+/−) mice (Tsunematsu *et al*., 2004) were treated with a single dose of urethane (in PBS; at 1 g·kg^−1^ body weight) by intraperitoneal injection at age of 8–9 weeks and sacrificed 40 weeks later for analysis of lung tissues. Lung tissues were fixed in 70% ethanol to permit tumor counting. Mice were bred and treated under the animal protocols that were approved by University of California at San Francisco (UCSF) Laboratory Animal Resource Center (LARC) or by Animal Welfare and Research Committee (AWRC) at Lawrence Berkeley National Laboratory (LBNL).

### 2.2 Cell lines and cell culture

The human NSCLS cell lines PC9 and H1299 were purchased from the Type Culture Collection of the Chinese Academy of Sciences (Shanghai, China). Gefitinib‐resistant (GR) HCC827GR and H3255GR cells together with their parental counterparts HCC827 and H3255 were kind gifts from Xiaojuan Wu. HCC827, PC9, and H1299 were cultured in RPMI 1640 supplemented with 10% FBS (BI; Invitrogen, Carlsbad, CA, USA). H3255 cell lines were maintained in BEBM supplemented with 10% FBS (Gibco, Invitrogen, Waltham, MA, USA). The GR cell lines were maintained in the presence of gefitinib (1 μm). All cells were grown in a humidified incubator at 37 °C with 5% CO_2_/95% air atmosphere and were revived every 3–4 months.

### 2.3 Establishment of FBXW7 stable knockdown cell lines using RNA interference

The lentiviral constructs expressing human FBXW7 short hairpin RNA were prepared using the pLVTHM‐GFP lentiviral RNAi expression system. The shRNA for FBXW7 is as follows:

shFBXW756: ccAGAGACTGAAACCTGTCTActcgagTAGACAGGTTTCAGTCTCTGG;
shFBXW758: ccAGAGAAATTGCTTGCTTTActcgagTAAAGCAAGCAATTTCTCTGG.

HCC827 and H1299 cells were infected with lentiviral particles containing specific or control vectors. Infected cells were then selected in media containing 2 μg·mL^−1^ puromycin for 48 h and after selection maintained in complete medium with 0.5 μg·mL^−1^ puromycin.

In PC9 cells, FBXW7 knockdown was achieved using a *FBXW7*‐specific siRNA. *FBXW7*‐specific siRNA (GCACAGAAUUGAUACUAACTT) and a negative control‐siRNA (UUCUCCGAACGUGUCACGUTT) were purchased from Invitrogen. For all transfection procedures, standard protocols were followed in accordance with manufacturer's instructions using Lipofectamine 2000 (Invitrogen).

### 2.4 Reagents and antibodies

Gefitinib (Selleckchem, Houston, TX, USA) and rapamycin (Solarbio, Beijing, China) were dissolved in DMSO at a stock concentration of 100 mm (stored at −80 °C) and then diluted in appropriate culture medium before used. Antibody against FBXW7 was purchased from Abcam (Cambridge, MA, USA). Phospho‐specific antibody against P70S6K was from Proteintech (Rosemont, IL, USA). β‐Actin, E‐cadherin, ZO‐1, vimentin, N‐cadherin, ZEB‐1, phospho‐mTOR, mTOR, and P70s6k antibodies were from Cell Signaling Technology (Danvers, MA, USA). Unless otherwise noted, all other chemicals were from Sigma‐Aldrich (St. Louis, MO, USA).

### 2.5 Western blotting

Cell lysates were prepared in RIPA buffer (1% Triton X‐100, 0.1% SDS, 50 mm Tris PH 7.5, 150 mm NaCl, 0.5% sodium deoxycholate, 10 mm NaF) supplemented with protease inhibitors (Roche, Indianapolis, IN, USA) and phosphatase inhibitors (Solarbio). Equal amounts of protein extracts were separated by 8–12% SDS/PAGE and then electroblotted to poly(vinylidene difluoride) membranes. After blocking with 5% (w/v) BSA in TBST (mixture of Tris‐buffered saline and Tween‐20) for 2 h at room temperature, the membranes were incubated with primary antibodies at 4 °C overnight (> 16 h). Subsequently, the membranes were incubated for 1 h at room temperature in a 1 : 10000 dilution of horseradish peroxidase‐conjugated secondary antibody (Sigma‐Aldrich) and visualized with chemiluminescence (Pierce Protein Biology Products/Thermo Scientific, Rockford, IL, USA).

### 2.6 Wound‐healing assay

Monolayer cells grown to confluence in six‐well plates were scratched using a sterile plastic 200‐μL micropipette tips. Next, the cells were washed three times with D‐Hanks and then cultured with serum‐free medium for 24 h before capture of images. Photographs focusing on the same position were taken immediately and at 24 h after wound incision with a phase‐contrast microscopy. Five areas were measured in each experiment.

### 2.7 Motility and invasion assay

A total of 5 × 10^4^ cells were seeded on BD Falcon Cell Culture Inserts with or without a thin layer of MATRIGEL Basement Membrane Matrix. The inserts were then placed on 24‐well plates containing complete serum as chemo‐attractant. After incubation in serum‐free medium for appropriate time (16 h for H1299 migration assay, 24 h for H1299 invasion assay, 38 h for HCC827 migration assay, and 48 h for HCC827 invasion assay), inserts were washed with PBS, fixed with 4% formalin (Sigma), and stained with Giemsa staining solution (Invitrogen). The unmigrated cells on the surface of the membrane were removed using cotton swabs. Images were taken and analyzed with imagej (National Institutes of Health, Bethesda, MD, USA) to acquire cell numbers.

### 2.8 Cell viability assay

Cells were plated onto 96‐well plates in sextuplicates at a density of 6 × 10^3^ per well with or without gefitinib/rapamycin treatment. The viability of cells was determined using 3‐(4, 5‐dimethylthiazol‐2‐yl)‐2, 5‐diphenyltetrazolium bromide (MTT) at the indicated time points. In brief, the MTT assay was performed by adding 10 μL MTT (5 mg·mL^−1^) into each well for 4 h after which particles were dissolved using DMSO. After full dissolution of the particles, the absorbance was measured at 492 nm on a microplate reader (Dynex Technologies, Chantilly, VA, USA).

### 2.9 *In vivo* tumor growth

BALB/c nude mice were purchased from HFK Bioscience Company (Beijing, China) and bred under specific pathogen‐free conditions. All animals were used in accordance with institutional guidelines, and the current experiments were approved by the Use Committee for Animal Care of Shandong University. For subcutaneous inoculation, HCC827 ctrl and HCC827 sh*FBXW7* 58 cells (3 × 10^6^) were, respectively, injected subcutaneously into the axilla of each nude mouse (4–6 weeks old). Four days after tumor inoculation, 20 mice were divided randomly into the following four groups that received either vehicle control (hanks), 5 mg·kg^−1^ gefitinib, 8 mg·kg^−1^ rapamycin, or 5 mg·kg^−1^ gefitinib combined with 8 mg·kg^−1^ rapamycin (*n* = 5 per group) intraperitoneally every 3 days for 21 days. Tumor size was measured every 3 days after tumors appeared, and the tumor volume was calculated by the formula length × width^2^/2.

### 2.10 Statistical analysis

spss 17.0 software (SPSS, Chicago, IL, USA) was used for statistical analysis. Statistical differences between two groups were assessed with the Student's *t*‐test and multiple groups with ANOVA test. *P *<* *0.05 was considered statistically significant. All results were expressed as mean ± SD from at least three independent experiments.

## 3 Results

### 3.1 Deficiency of *Fbxw7* is susceptible to lung tumor development in mice

**Figure 1:**
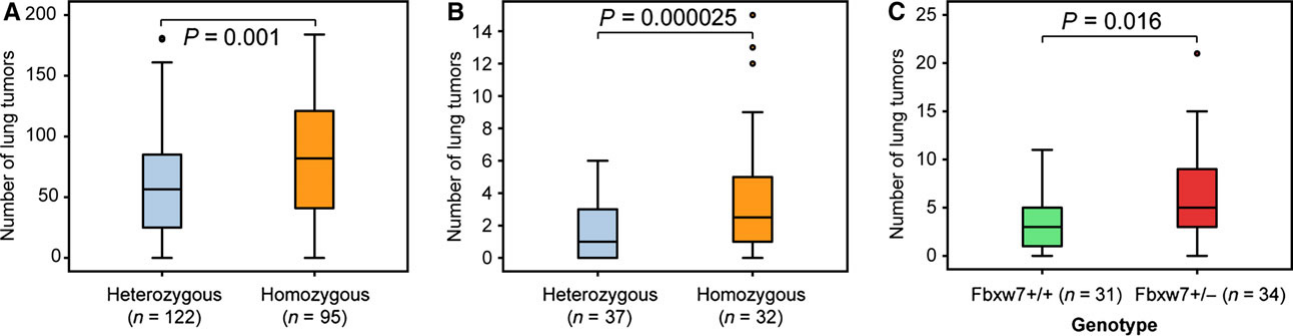
*Fbxw7* deficiency is susceptible to lung cancer development in mice. (A, B) Fbxw7 polymorphism (Asp61Asn of Fbxw7α) is associated with lung cancer development in mouse F1 backcross (F1Bx) studies: (A) F1Bx between SPRET/EiJ and FVB/N *Kras2* ^*LA*^ ^*2*^ mice, and (B) F1Bx between SPRET/EiJ and FVB/N mice where lung tumors were induced by a single treatment of urethane. (C) Loss of a single copy of Fbxw7 significantly increases urethane‐induced lung tumor development: box‐plot of lung tumors in *Fbxw7*+/− and wild‐type mice (*P* = 0.016).

To assess whether *Fbxw7* plays a role in lung cancer development, we first carried out a genetic analysis of the *Fbxw7* locus in our two previous mouse backcross studies: the first one was between SPRET/EiJ and FVB/N*Kras2* ^*LA2*^ mice where lung tumor development was initiated by knock‐in of mutant K‐ras (G12D), and the second was between SPRET/EiJ and FVB/N mice where lung tumor development was induced by a single treatment of urethane (To *et al*., 2006). In both lung carcinoma models, mice that inherited one SPRET/EiJ *Fbxw7* allele (Asp61 of Fbxw7α) developed significantly fewer lung tumors compared to mice homozygous for the FVB/N allele (Asn61 of Fbxw7α) (Fig. 1A,B), consistent with our previous findings that the SPRET/EiJ *Fbxw7* allele confers resistance to tumor development (Perez‐Losada *et al*., 2012).

In order to confirm whether deficiency of *Fbxw7* increases susceptibility to lung cancer development induced by urethane, 34 *Fbxw7* heterozygous (*Fbxw7*+/−) mice, which was described in Tsunematsu *et al*. (2004), and 31 *Fbxw7* wild‐type mice were injected intraperitoneally with a single dose of urethane. After 40 weeks, all mice were sacrificed and the number of lung tumors counted. We observed a statistically significant increase in the number of lung tumors in *Fbxw7+*/− mice (Fig. 1C) in agreement with the hypothesis that this gene is a general tumor suppressor in multiple tissues.

### 3.2 Reduced FBXW7 expression correlates with poor disease outcome

**Figure 2:**
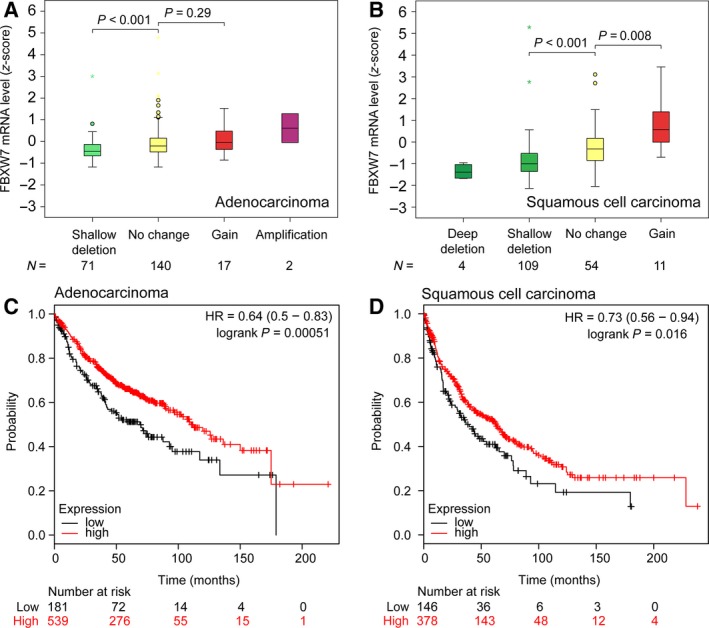
Reduced expression of *FBXW7* is associated with poor prognosis of human lung cancer patients. (A, B) Expression of FBXW7 is positively correlated with its copy number changes in lung adenocarcinomas (A) and squamous cell carcinomas (B) using The Cancer Genome Atlas datasets. (C, D) Low expression of FBXW7 significantly decreases overall survival of lung adenocarcinomas (C) and squamous cell carcinomas (D) patients using KM plotter analysis (http://kmplot.com/analysis/index.php?p=service&cancer=lung).

To assess the roles of *FBXW7* in human lung cancer development, we first examined DNA copy number changes of *FBXW7* in lung adenocarcinoma (AC) and squamous cell carcinoma (SCC) using The Cancer Genome Atlas data and found that deletion of *FBXW7* was found in 71 of 230 (30.9%) ACs and 113 of 178 (63.5%) SCCs, while gain of *FBXW7* was only found 19 (8.3%) ACs and 11 (6.2%) SCCs (Table S1). Moreover, 4 (1.7%) ACs and 11 (6.2%) SCCs contained a mutation of *FBXW7* (Table S1), which is similar to the frequency reported in the Catalogue Of Somatic Mutations In Cancer (COSMIC) database (http://cancer.sanger.ac.uk/cosmic). The deletion of *FBXW7* significantly led to reduction in FBXW7 mRNA expression in both ACs and SCCs (Fig. 2A,B). These results indicated that genomic loss of FBXW7 DNA copy number is one of the main mechanisms through which gene expression is reduced in human lung cancer. To determine whether reduced FBXW7 expression is associated with clinical outcome of lung cancer patients, we further evaluated the prognostic value of *FBXW7* in a large public clinical microarray database (Gyorffy *et al*., 2013). Kaplan–Meier analysis showed that tumors with high expression of *FBXW7* had significant longer overall survival in both AC and SCC (Fig. 2C,D). Together, these clinical data indicate that the expression levels of *FBXW7* are reduced during lung cancer development and that reduced *FBXW7* expression correlates with poor clinical outcome.

### 3.3 FBXW7 knockdown induces EMT in NSCLC cells

**Figure 3:**
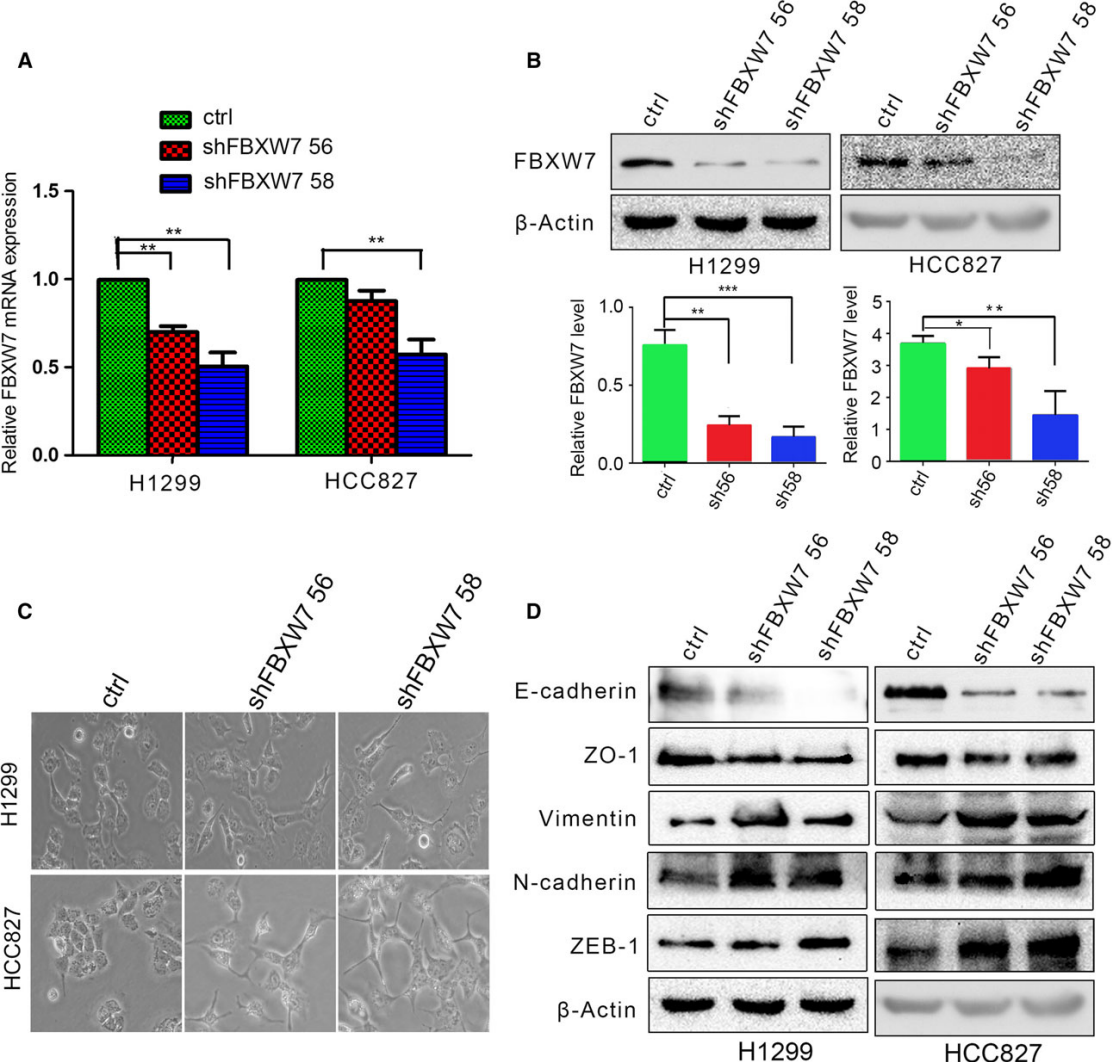
FBXW7 loss induces EMT in NSCLC. (A, B) The reduced mRNA (A) and protein (B) levels of FBXW7 were measured by qRT–PCR and western blotting in H1299 and HCC827 cell lines stably transfected with shFBXW7 56 and shFBXW7 58. The graph shows quantitative analysis. (C) Representative micrographs of H1299‐shFBXW7 and HCC827‐shFBXW7 cells under bright field display significant differences in morphology. (D) Expressions of epithelial markers (E‐cadherin and ZO‐1) and mesenchymal markers (vimentin, N‐cadherin, and ZEB‐1) were analyzed by western blotting. All data are presented by mean ± SD. *P < 0.05, **P < 0.01, ***P < 0.001 based on the Student's *t*‐test. All results are from three independent experiments.

To examine whether FBXW7 regulates epithelial–mesenchymal transition (EMT) in NSCLC cells, we knocked down FBXW7 using two different short hairpin RNA (shRNA) constructs (shFBXW7 56 and shFBXW7 58) into H1299 cells and HCC827 cells. The levels of FBXW7 in these resultant cell lines were verified by qRT–PCR and western blotting (Fig. 3A,B). Compared to the control cells, morphologic changes in FBXW7 knockdown cells were observed with a discohesive growth pattern and a spindle‐shaped fibroblastic appearance, the typical features of cells undergoing EMT (Fig. 3C). Consistent with these observations, western blotting revealed obvious losses of epithelial markers (E‐cadherin and ZO‐1), accompanied by apparent increases in mesenchymal markers (vimentin, N‐cadherin, and ZEB‐1) in both HCC827‐shFBXW7 cells and H1299‐shFBXW7 cells (Fig. 3D). These findings suggest that FBXW7 knockdown promotes EMT in NSCLC cells.

### 3.4 FBXW7 inhibits migration and invasion of NSCLC cells

**Figure 4:**
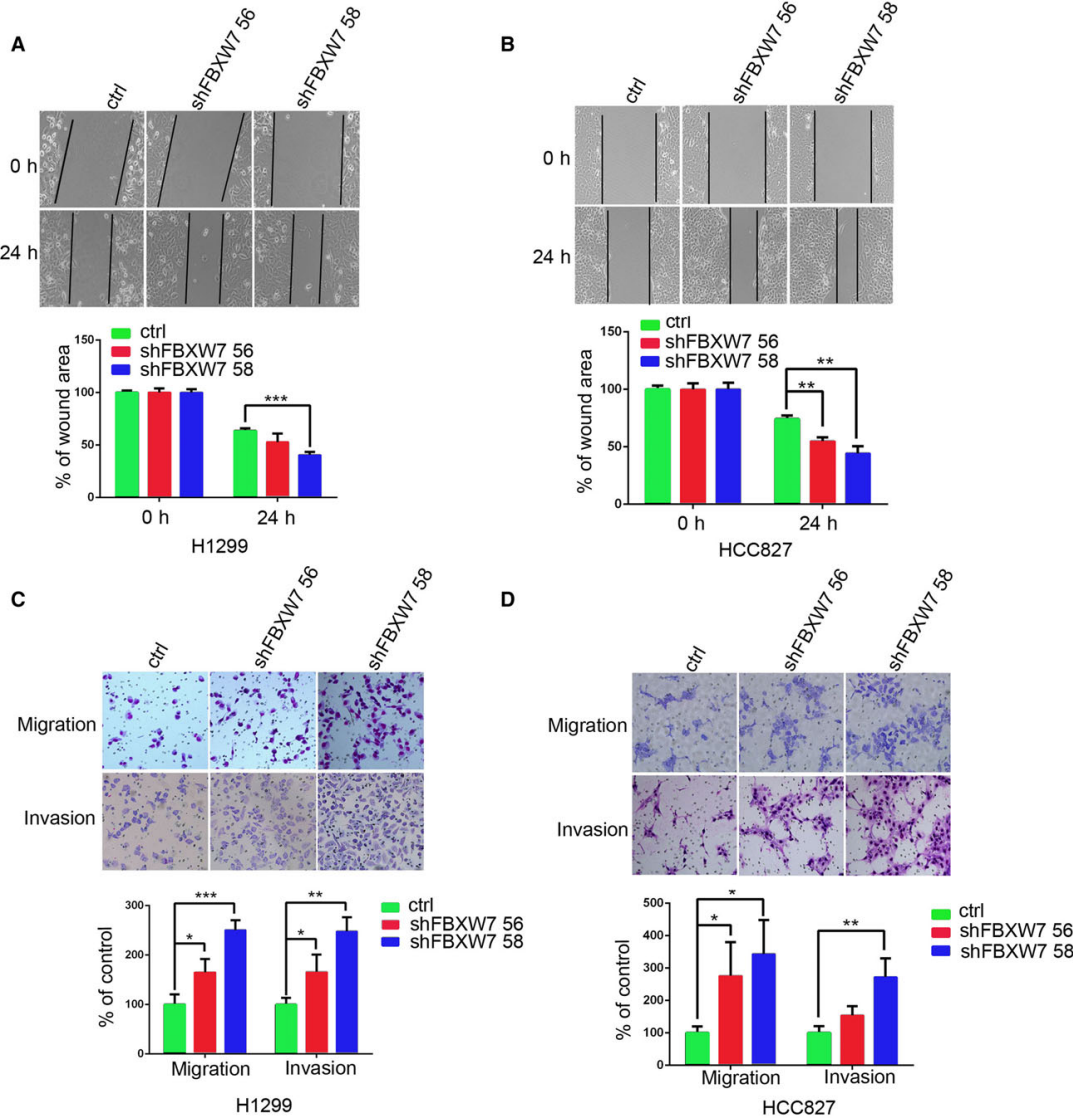
FBXW7 inhibits NSCLC cell migration and invasion. (A, B) H1299‐shFBXW7 (A) and HCC827‐shFBXW7 cells (B) or their control vector cells were subjected to wound‐healing assay. The graph shows quantitative analysis (bottom). (C, D) H1299‐shFBXW7 (C) and HCC827‐shFBXW7 cells (D) or their control vector cells were subjected to Transwell migration and Matrigel invasion assays. The graph shows quantitative analysis (bottom). Data are presented as mean ± SD. *P < 0.05, **P < 0.01, ***P < 0.001 based on the Student's *t*‐test. Results in A–D are from three independent experiments.

Next, we assessed the function of FBXW7 on EMT associated cell behaviors in NSCLC cells. First, we performed the wound‐healing assay to examine the effect of FBXW7 on cell migration. We found that both HCC827‐shFBXW7 cells and H1299‐shFBXW7 cells had significantly faster closure of the wound area compared to their respective control cells (Fig. 4A,B). This result was confirmed using the Boyden chamber‐based cell migration assay (Fig. 4C,D). Additionally, both HCC827‐shFBXW7 cells and H1299‐shFBXW7 cells harbored enhanced invasiveness compared to their control cells in the Matrigel invasion chamber assay (Fig. 4C,D). These data indicate that FBXW7 inhibits migratory and invasive capabilities of NSCLC cells.

### 3.5 FBXW7 knockdown leads to gefitinib resistance *in vitro*

**Figure 5:**
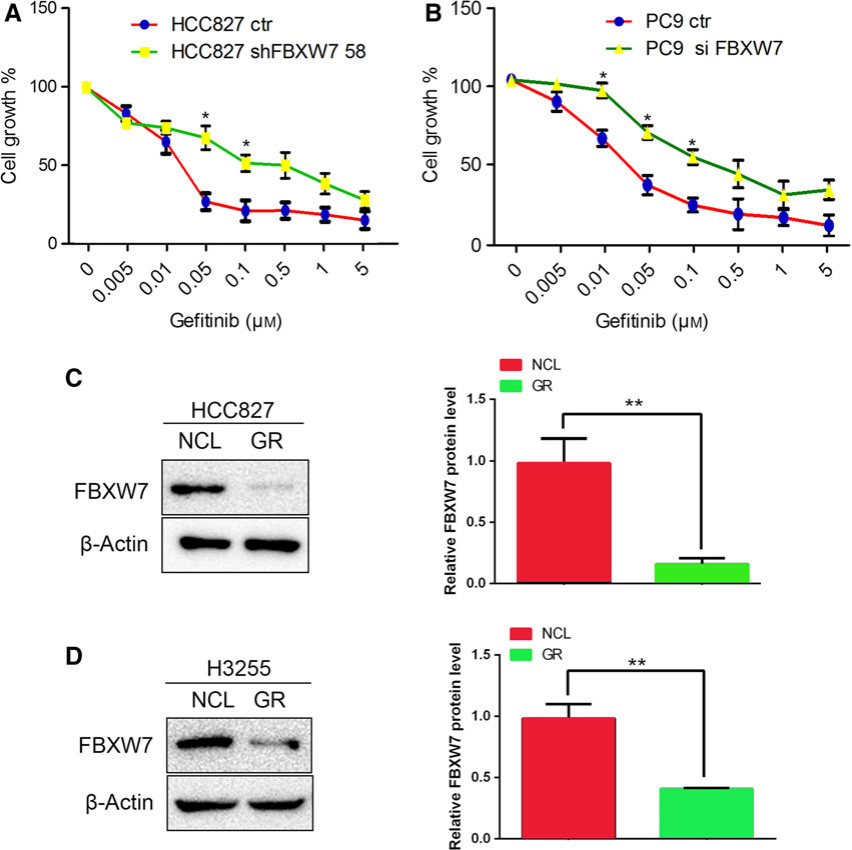
FBXW7 downregulation induces gefitinib resistance *in vitro*. (A, B) HCC827‐shFBXW7 58 (A) and PC9‐siFBXW7 cells (B) or their control cells were treated with gefitinib at indicated concentration in 96‐well plates for 72 h, and the cell viability was examined by MTT assays. (C, D) FBXW7 expression was detected in HCC827GR (C) and H3255GR (D) cell lines by western blotting. NCL, normal cell line; GR, gefitinib resistant. The graph shows quantitative analysis. Data are presented as mean ± SD. *P < 0.05, **P < 0.01 based on the Student's *t*‐test. All results are from three independent experiments.

Increasing evidence shows a correlation between FBXW7 expression and drug resistance. Therefore, we investigated whether FBXW7 had an impact on gefitinib sensitivity in NSCLC cells. First, HCC827‐shFBXW758, H1299‐shFBXW758, and their control cells were treated with different concentrations of gefitinib for 72 h. The inhibitory effect of gefitinib on the growth of HCC827, a gefitinib‐sensitive cell line, was significantly impaired after FBXW7 knockdown while no significant change was observed in GR H1299 cells (Fig. 5A and Fig. S1). We then tested the influence of FBXW7 on the efficacy of gefitinib in another gefitinib‐sensitive PC9 cells, which harbor EGFR (delE746‐A750) mutation. The expression of FBXW7 in PC9 cells was transiently silenced using FBXW7‐specific siRNA, which was confirmed by qRT–PCR and western blotting (Fig. S2A,B). Subsequent cell growth analysis revealed that, similar to HCC827, PC9 cells with FBXW7‐siRNA transfection exhibited increased gefitinib resistance compared to PC9 cells transfected with control‐siRNA (Fig. 5B). These data indicate that knockdown of FBXW7 reduced TKI sensitivity in EGFR‐mutated cell lines to some extent.

To confirm the involvement of FBXW7 in gefitinib resistance, FBXW7 expression was determined in acquired GR HCC827GR and H3255GR cells. Interestingly, both HCC827GR and H3255GR cells expressed lower levels of FBXW7 compared to their parental gefitinib‐sensitive counterparts (Fig. 5C,D). Collectively, these results indicate that FBXW7 positively mediates therapeutic efficacy of gefitinib in EGFR‐mutated NSCLC cell lines.

### 3.6 Rapamycin eliminates gefitinib resistance induced by FBXW7 knockdown

**Figure 6:**
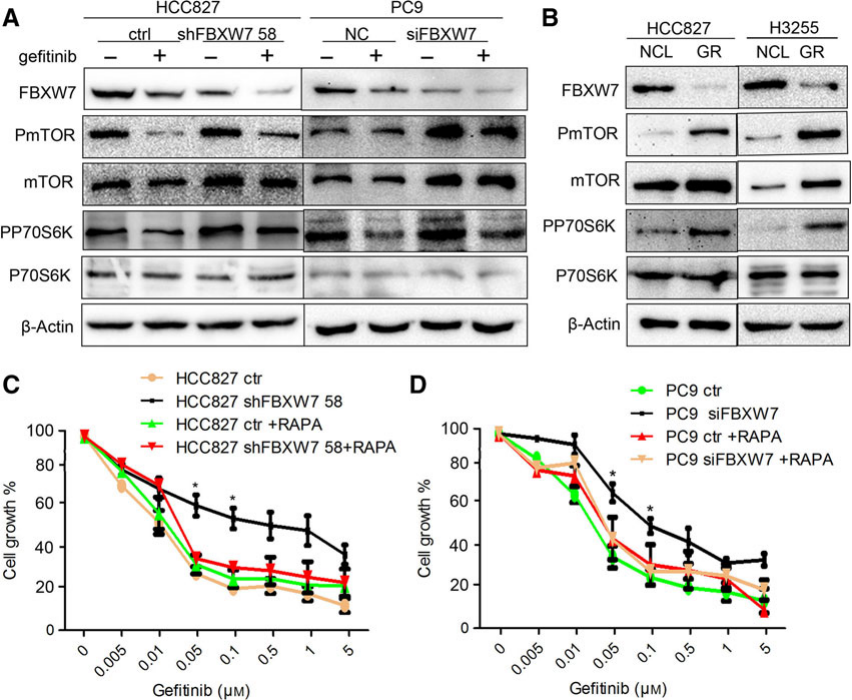
FBXW7 influences gefitinib sensitivity by targeting mTOR pathway. (A) Levels of FBXW7, mTOR, PmTOR, P70S6K, PP70S6K were analyzed by western blotting in HCC827‐shFBXW7 58 and PC9‐siFBXW7 cells or their control cells with or without gefitinib treatment. (B) HCC827GR and H3255GR lysates along with their control cell lysates were subjected to western blotting analysis and probed with the above antibodies. (C, D) The cell viability of HCC827‐shFBXW758 (C) and PC9‐siFBXW7 cells (D) or the control cells was measured by MTT assays following the treatment of gefitinib, rapamycin, or the combination of gefitinib and rapamycin for 72 h. Data are reported as mean ± SD. *P < 0.05. All data are based on three independent experiments.

As mTOR is one of the downstream substrates of FBXW7 and a case study of a lung cancer patient harboring an FBXW7 mutation reported clinical and radiographic benefit from treatment with an mTOR inhibitor (Villaruz and Socinski, 2014), we addressed the possibility that FBXW7 executed its role in gefitinib treatment through interfering mTOR signaling pathway. As indicated in Fig. 6A, in HCC827 and PC9 control cell line, the phosphorylation of mTOR and its downstream protein P70S6K was markedly reduced upon gefitinib treatment. In contrast, in the FBXW7 silencing HCC827‐shFBXW7 cells and PC9‐siFBXW7 cells, the total protein level of mTOR along with phosphorylation of mTOR and P70S6K was dramatically increased and persisted at higher levels with or without gefitinib treatment. Moreover, accompanied by attenuated FBXW7 level, HCC827GR cells and H3255GR cells had elevated mTOR, phosphor‐mTOR, and phosphor‐P70S6K levels compared to their parental cells (Fig. 6B).

To determine whether increased mTOR signaling underlies the gefitinib resistance induced by FBXW7 depletion, we examined whether mTOR inhibition suppressed growth of the resistant cells. HCC827‐shFBXW7 cells and PC9‐siFBXW7 cells were exposed to rapamycin, a mTOR signal inhibitor in combination with gefitinib. Combined treatment successfully reversed drug resistance of HCC827 and PC9 cells triggered by loss of FBXW7 expression (Fig. 6C,D).

**Figure 7:**
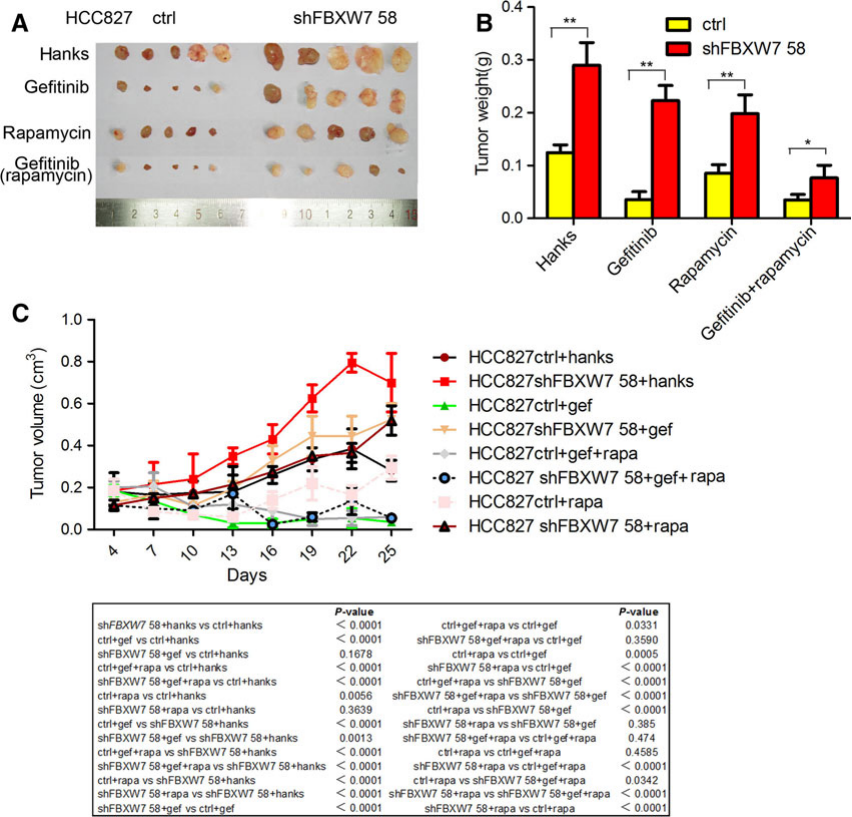
Rapamycin reverses FBXW7 knockdown induced gefitinib resistance *in vivo*. (A) FBXW7 silenced HCC827 cells and their corresponding control cells were subcutaneously injected into nude mice (*n* = 5) with treatments described in Section 2. After 25 days, the nude mice were sacrificed. The images of the dissected tumors are shown. A ruler is used to demonstrate the size of the tumor. (B) The body weight of mice with different treatment was presented. (C) The volumes of the subcutaneous tumors were measured every 3 days from the 4th day following implantation. The comparisons of statistics were carried out with ANOVA test (bottom). Data are represented as mean ± SD. *P < 0.05, **P < 0.01, based on the Student's *t‐*test.

To further confirm our *in vitro* findings, HCC827‐shFBXW7 and its control cells were subcutaneously inoculated into athymic mice. Consistent with the *in vitro* data, downregulation of FBXW7 accelerated tumor formation at the implantation site and promoted gefitinib resistance *in vivo*. In contrast, the tumors originating from HCC827 control cells grew less rapidly and appeared to be sensitive to gefitinib treatment. Fortunately, the suppressive effect of gefitinib was intensified when combined with rapamycin even for the refractory HCC827‐shFBXW7 cells (Fig. 7A–C). Taken together, we conclude that FBXW7 contributes to the efficiency of gefitinib treatment by interfering with the mTOR signaling pathway.

## 4 Discussion

EGFR‐TKI has been recommended as the first choice for advanced NSCLC patients with EGFR mutation on account of the fact that it can bring about longer progression‐free survival, less severe adverse events and prolongs survival in NSCLC patients who fail the chemotherapy regimens (Mitsudomi *et al*., 2010; Rosell *et al*., 2012; Shepherd *et al*., 2005). However, drug resistance ultimately develops. Although several systemic treatment options have been established for drug‐resistant NSCLC patients, more efforts should be made to conquer such challenges (Fruh, 2011).

Recent studies have identified the mechanisms associated with resistance to EGFR‐TKI in NSCLC. Among those resistance mechanisms, the secondary EGFR mutation, T790M in exon 20 of the EGFR gene, plays the most significant role and arises in ~ 60% of the cases (Sun *et al*., 2013; Sutto and Gervasio, 2013; Yun *et al*., 2008). To target this mutation, reversible indolocarbazole‐based or irreversible pyrimidine‐based TKIs, such as WZ4002, AZD9291, and CO‐1686, have been used in recent preclinical studies (Cross *et al*., 2014; Lee *et al*., 2013; Walter *et al*., 2013; Zhou *et al*., 2009). Other mechanisms explaining EGFR‐TKI resistance include the primary resistance driven by K‐RAS mutation, activation of PI3K/AKT pathway, NF‐κB activation, EML4‐ALK gene rearrangements, EMT involvement as well as acquired resistance caused by MET amplification, loss or decreased DNA copy number of the activating EGFR mutant gene and genotypic and histological transformation from NSCLC into small cell lung cancer (Bivona *et al*., 2011; Coldren *et al*., 2006; Engelman *et al*., 2006; Sequist *et al*., 2011; Shaw *et al*., 2009; Tabara *et al*., 2012; Turke *et al*., 2010; Zhu *et al*., 2008). In the present study, we emphasize the function of FBXW7 in controlling of EGFR‐TKI resistance in NSCLC.

FBXW7, a member of F‐box family proteins, is a substrate recognition component of the SCF (complex of SKP1, CUL1, and F‐box protein) E3 ubiquitin ligase complex that mediates the ubiquitin‐dependent proteolysis of several oncoproteins, such as MCL1, Myc, cyclin E, and mTOR, so it is regarded as a tumor suppressor at the crossroads of cell division, growth, and differentiation (Cheng and Li, 2012; Mao *et al*., 2008; Welcker and Clurman, 2008). Low FBXW7 expression is associated with decreased chemotherapeutic sensitivity, like taxol and cisplatin in NSCLC (Yokobori *et al*., 2014; Yu *et al*., 2013). Moreover, Wertz *et al*. (2011) suggested profiling the FBXW7 and MCL1 status of tumors, in terms of protein levels, messenger RNA levels along with genetic status, could be of great use in predicting the response of patients to antitubulin chemotherapeutics. In addition, MCL1 accumulation caused by *FBXW7* mutation in SCC led to resistance to the BH3 mimetic ABT‐737 but enhanced synergistic effects of combination the HDAC inhibitor vorinostat with ABT‐737 (He *et al*., 2013).

As one of the established causes of drug resistance, EMT can adversely promote cancer progression through endowing cells with enhanced migratory, invasive, anti‐apoptotic combined with drug‐resistant properties, and hence, profiling important EMT markers could imply chemosensitivity and tumor prognosis (Hoshino *et al*., 2009; Jechlinger *et al*., 2003; Kurrey *et al*., 2009; Nozawa *et al*., 2006). In addition, the enzyme mTOR functions as a serine/threonine protein kinase that regulates cell growth, proliferation, motility, survival, protein synthesis, autophagy as well as cell cycle progression, and mTOR pathway activation leads to tumor development (Hay and Sonenberg, 2004). Previous studies have indicated that targeting mTOR can enhance apoptosis and increase sensitivity to chemotherapeutics. Furthermore, mTOR inhibitors manage to overcome EGFR‐TKI resistance in NSCLC (Fei *et al*., 2013; Ishikawa *et al*., 2013; Shi *et al*., 1995).

Here, we focus on FBXW7, a bona fide tumor suppressor that restrains EMT and directly target mTOR for ubiquitin degradation in NSCLC. Not only do we demonstrate the imperative role of FBXW7 in control of EMT and NSCLC cell invasion, but also its pivotal responsibility to guarantee effectiveness of gefitinib via inhibiting mTOR/p70S6K pathway in EGFR‐mutated NSCLC. Additionally, in the condition that mTOR signaling is aberrantly activated to abrogate gefitinib‐induced cell growth inhibition because of FBXW7 downregulation, the administration of rapamycin is sufficient to reverse gefitinib resistance, leading to the firmer conviction that FBXW7 plays a crucial role mediating EGFR‐TKI sensitivity via mTOR/p70S6K pathway (Fig. S3).

## 5 Conclusion

Our studies focus on the views that FBXW7 executes its tumor suppressor function by inhibition of EMT and the mTOR/p70S6K pathway, ensuring the efficiency of EGFR‐TKI treatment in NSCLC. This finding suggests that FBXW7 may serve as a potential molecular marker for predicting EGFR‐TKI treatment response and prognosis in NSCLC patients with EGFR mutation and, more importantly, provides a new treatment strategy for these patients.

## Author contributions

PZ and JHM designed the study. XY, YC, WY, LH, WW, HY, JPL, and AMS performed the experiments and the data analysis. XY wrote the manuscript. PZ, AMS, and JHM edited the manuscript. All authors read and approved the final manuscript.

## Supporting information


**Fig. S1.** FBXW7 downregulation does not influence gefitinib sensitivity of H1299 cells.
**Fig. S2.** FBXW7 expression is silenced in PC9 cells by siFBXW7.
**Fig. S3.** Schematic diagram of FBXW7‐mTOR signaling axis in the regulation of tumorigenesis and EGFR‐TKI sensitivity of NSCLC.

**Table S1.** FBXW7 alteration in lung adenocarcinomas and squamous cell carcinomas from TCGA study.
