# Supplementary material for: FBXW7 deletion contributes to lung tumor development and confers resistance to gefitinib therapy
Source: Mol Oncol. 2018 May 9;12(6):883–95. doi: 10.1002/1878-0261.12200 (PMC5983212; doi:10.1002/1878-0261.12200)
Supplement: Supplementary file 1 — Fig. S1. FBXW7 downregulation does not influence gefitinib sensitivity of H1299 cells. Fig. S2. FBXW7 expression is silenced in PC9 cells by siFBXW7. Fig. S3. Schematic diagram of FBXW7‐mTOR signaling axis in the regulation of tumorigenesis and EGFR‐TKI sensitivity of NSCLC. [file MOL2-12-883-s001.pdf]

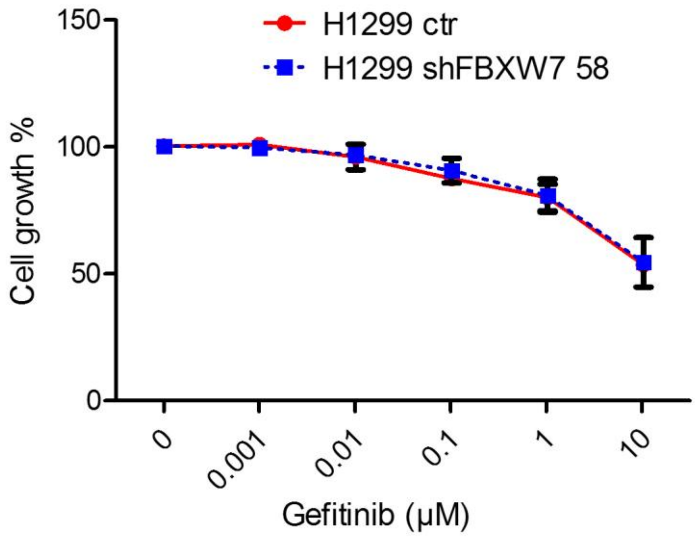

Supplementary Fig. 1. FBXW7 downregulation does not influence gefitinib sensitivity of H1299 cells. H1299-shFBXW7 58 cells and their control cells were treated with gefitinib at indicated concentration in 96-well plates for 72h and the cell viability was examined by MTT assays.

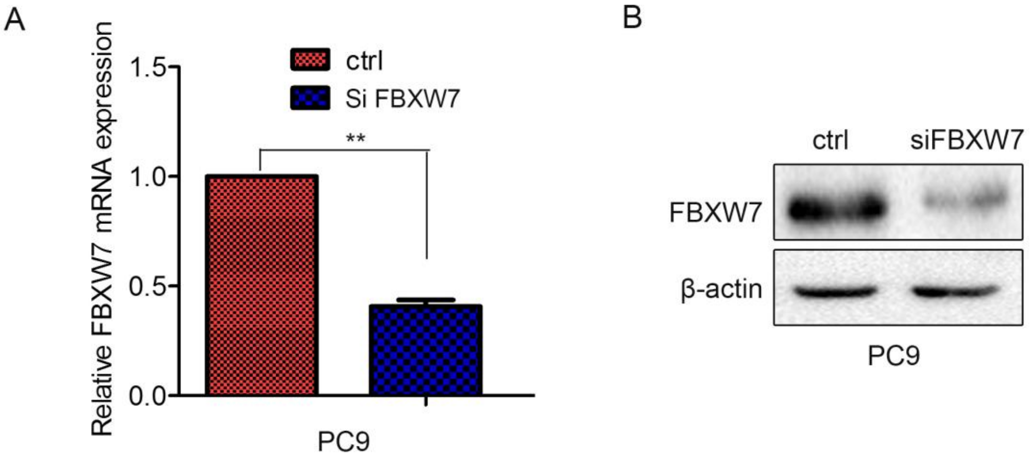

Supplementary Fig. 2. FBXW7 expression is silenced in PC9 cells by siFBXW7. (**A and B**) The reduced mRNA (A) and protein (B) levels of FBXW7 were measured by qRT-PCR and Western blotting in PC9 cell lines transiently transfected with siFBXW7.

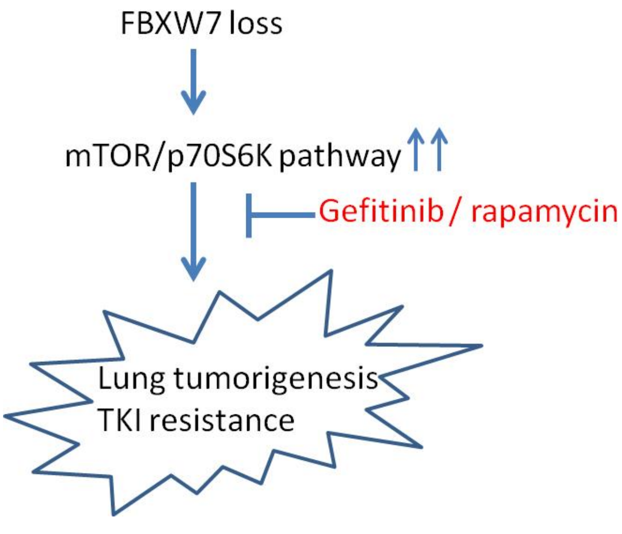

Supplementary Fig. 3. Schematic diagram of FBXW7-mTOR signaling axis in the regulation of tumorigenesis and EGFR-TKI sensitivity of NSCLC.
